# A Survey of Cannabis Acute Effects and Withdrawal Symptoms: Differential Responses Across User Types and Age

**DOI:** 10.1089/acm.2018.0319

**Published:** 2019-03-09

**Authors:** Michelle Sexton, Carrie Cuttler, Laurie K. Mischley

**Affiliations:** ^1^ Department of Anesthesiology, University of California San Diego, San Diego, CA.; ^2^ Department of Psychology, Washington State University, Pullman, WA.; ^3^ Research Institute, Bastyr University, Kenmore, WA.

**Keywords:** neuropsychological effects, phytocannabinoids, cognitive impairment, medical cannabis

## Abstract

***Objectives:*** There is a rapidly evolving legal and medical culture around cannabis, with corresponding changes in the demographics of users. For instance, the percentage of the aging population accessing cannabis is growing substantially, outpacing other age groups. The goals of this study were to describe the acute effects of cannabis, subjective experiences of withdrawal, and beliefs around the addictiveness of cannabis, as well as to determine whether these effects differ as a function of age or reason for use (medical vs. recreational use). It was hypothesized that medical users and younger users would report fewer adverse effects.

***Subjects:*** Survey responses from 2905 cannabis users were analyzed.

***Results:*** Hierarchical logistic regression analyses were used to compare group percentages after statistically controlling for confounding differences in their demographic and cannabis use characteristics. The most commonly endorsed acute effects were improved sleep, more calm/peaceful, desire to eat, more creative, and dry mouth; while the most commonly endorsed withdrawal symptoms were irritability, insomnia, and anxiety. Relative to recreational users, medical users were less likely to report undesirable acute effects but were more likely to report undesirable withdrawal symptoms. Older (50+) individuals reported fewer undesirable acute effects and withdrawal symptoms compared with younger users (18–29). Only 17% of the total sample reported believing that cannabis is addictive, and this did not vary as a function of reason for use.

***Conclusions:*** Older people and medical users appear to experience acute and withdrawal effects of cannabis differently than recreational and younger users, perhaps because these groups benefit more from the medicinal properties of cannabis. These data can provide descriptive information to help inform health care providers and potential consumers about effects of cannabis use.

## Introduction

Individuals previously naïve to *Cannabis* sp. may explore its medical application for a variety of symptoms, diagnoses or for weaning from prescription drugs such as opioids.^1–5^ Further, trends toward the legalization of adult use of cannabis are sparking renewed interest in the acute psychotropic effects of cannabis. Prevalence of cannabis use among the 50+ age group, from an ongoing cross-sectional cohort in the United States (*n* = 47,140), increased from 2.8% to 4.8% between 2006 and 2013.^6^ The changing demographic of cannabis users calls for the need to further evaluate benefits and risks in different user types and age groups. For instance, the potential for cannabis to impair cognition, memory, and balance may cause concern about potential health harms in older individuals and/or medically compromised individuals who may already be experiencing some of these symptoms. This leads to a nondirectional hypothesis that older and medical users would report different effects from younger and recreational users. The psychotropic effects profile may be the primary limiting factor in employing cannabis as a medicine.^7^

Some cannabis drug effects may be experienced acutely, others continue with ongoing use or emerge over time, and tolerance may develop to certain effects.^8–12^ Heterogeneity across studies impacts the interpretation of findings; however, there is a strong level of evidence for acute and chronic effects of cannabis on cognition, with mixed evidence of recovery of function upon abstinence.^13–15^ In addition, there are gender differences in the experience of both acute effects and withdrawal symptoms.^16^ Anxiety and paranoia are other potential side effects of cannabis; yet a survey reported that 51.8% of medical users used cannabis to treat anxiety, and another recent report suggests that acute cannabis intoxication decreases anxiety symptoms by 58%.^17–19^ This incongruence of reported effects may be confusing to new users.

Delta-9 tetrahydrocannabinol (THC) is a partial agonist (low intrinsic efficacy) at cannabinoid 1 (CB_1_) receptor, responsible for the psychoactive effects of cannabis.^20^ CB_1_ receptor is found at high levels in the following areas of the brain: cerebellum, hippocampus, basal ganglia, hypothalamus, and basolateral amygdala.^21^ These brain regions regulate motor function, posture, memory, appetite, and fear extinction. The effects of THC are mediated by release or reuptake inhibition of a variety of neurotransmitters.^22–25^ Cannabidiol (CBD) is a low-affinity antagonist of CB_1_ agonists (such as THC), thus lacking, or modulating, psychoactivity.^22,26^ CBD therapeutic properties include antipsychotic, anticonvulsant, and anxiolytic, while preclinical studies report antidepressant, anti-inflammatory, antineoplastic, and “alerting” effects.^27–40^ These actions are attributed primarily to binding at noncannabinoid receptors.^41,42^

Potential medical cannabis users and new adult users, previously naïve to the effects, are looking for information related to cost, administration forms, social stigma, and the experience of being “high.”^43^ An increasing number of individuals are turning to their doctors who may not be confident in providing information or guidance.^44–46^ Therefore, data from a large-scale cross-sectional survey of regular cannabis users were analyzed to document their subjective acute and withdrawal effects. The authors further aimed to examine differences in these effects as a function of age and type of use, hypothesizing that there may be differential effects. Finally, they report users' beliefs about the addictiveness of cannabis.

## Materials and Methods

### Participants

**FIG. 1.**
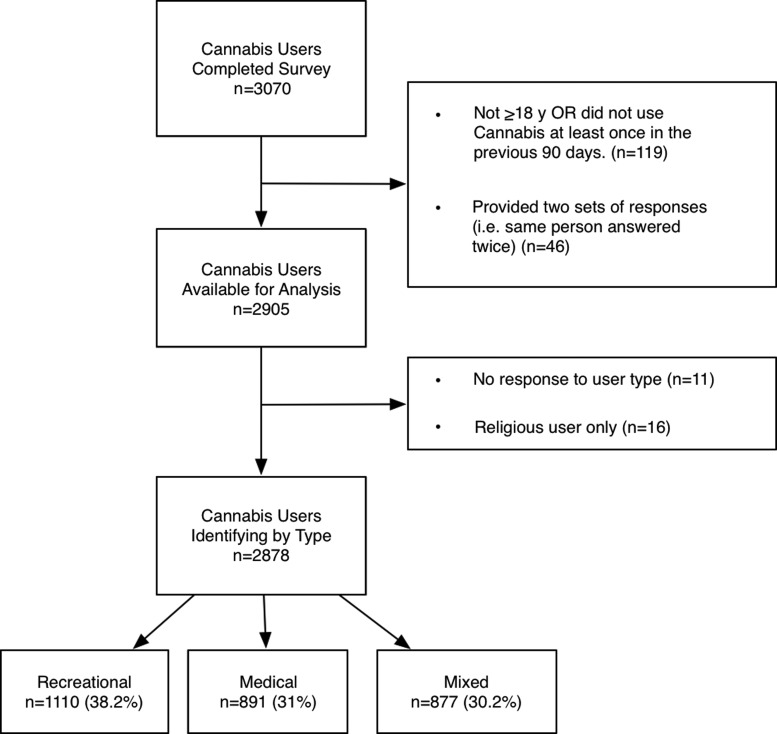
Flow diagram of participants.

A sample of 3070 participants was recruited through word of mouth, links on a variety of cannabis-related websites, and in Washington State cannabis retail outlets between December 2013 and January 2018. The only inclusion criteria were age ≥18 years and use of cannabis in the past 90 days. One hundred and nineteen respondents did not meet these criteria and were excluded. Forty-six respondents were identified as providing more than one set of responses, so data from their second set of responses were excluded. Therefore, the final sample comprised 2905 adult cannabis users. (Fig 1).

### Procedure and materials

Study data were collected and managed using Research Electronic Data Capture (REDcap), a secure tool allowing participants to directly enter responses. Bastyr University's Internal Review Board approved the protocol. Participants answered an anonymous online survey containing the items designed to assess cannabis use patterns and effects. No compensation was provided.

#### Survey questions

The potential acute effects (descriptors) were derived from the existing literature, and cannabis withdrawal symptoms were derived from the Diagnostic and Statistical Manual of Mental Disorders 5 (DSM-5).^47^ This list was circulated to medical professionals utilizing cannabis clinically in an iterative process. The researchers attempted to balance the number of positive and negative descriptors.

#### Demographic questions

Participants were asked to provide information about their age, gender, ethnicity, employment status, relationship status, highest level of education, and total family income. Response options for each are shown in Table 1.

**Table 1.** Sample Demographic Characteristics and Cannabis Use Patterns (*n* = 2905)

Gender, %		Ethnicity, %	
Male	53.4	Caucasian/White	84.3
Female	45.5	Black	1.8
Missing	1.1	Hispanic	3.8
Age		Native American	1.2
Range	18–80	Asian/Pacific Islander	1.6
Mean	34.96	Other	5.9
Standard deviation	13.67	Missing	1.5
Education, %		Income, %	
Less than high school	2.3	<$20,000	19.6
High school/GED	26.9	$20–40,000	23.4
Technical school	11.1	$40–60,000	15.9
Associate	14.9	$60–80,000	11.1
Bachelor's	29.6	$80–100,000	9.3
Master's	9.1	$100–150,000	9.9
Doctorate	5.1	>$150,000	7.7
Missing	1.1	Missing	3.1
Current employment, %		Relationship status, %	
Full-time	52.6	Single	40.1
Part-time	19.8	Married	32.6
Unemployed	12.6	Domestic	14.4
Retired	4.9	Divorced	5.5
Disabled	9.0	Other	6.4
Missing	1.2	Missing	0.9
Frequency of use, %		Method of use, %	
All day, everyday	9.2	Inhalation	91.3
5–10 times per day	12.1	Oral	7.4
1–4 times per day	42.2	Other (e.g., topical)	1.3
3–6 times per week	15.4	Method of selection, %	
1–3 times per week	10.3	High THC	41.4
2–3 times per month	5.2	High CBD	32.0
Once a month	2.0	Terpenoids	9.3
Less than once a month	3.6	Smell	43.9
Quantity (per week), %		Age of first use, %	
>1 oz (28 g)	1.8	<14	14.4
1 oz (28 g)	4.4	14–16	37.1
1/4 oz (7 g)	20.9	17–18	23.2
3–5 g	30.2	19–20	10.0
1–2 g	21.9	21–25	8.6
<1 g	20.9	>25	3.1

CBD, cannabidiol; THC, delta-9 tetrahydrocannabinol.

#### Cannabis use patterns

Participants indicated whether (yes/no) they use cannabis for recreational (adult use) purposes and/or medicinal purposes. They were asked to indicate the method of administration they most commonly use, their frequency of use, quantity of cannabis used per week, and the age they first used cannabis. They were also asked to indicate which of the following they consider important factors when selecting cannabis: high THC content, high CBD content, terpenoid content, and smell.

#### Acute effects

Participants were provided a list of 45 possible acute effects of cannabis (Table 2) and were asked to use a yes/no scale to indicate which immediate effects they experience (selecting as many as applied).

**Table 2.** Percentage of Total Respondents Endorsing Various Acute Effects of Cannabis

Cognitive, %		Psychological, %	
Sense of clarity/perspective	44.5	More calm/peaceful	79.7
Short-term memory problems	42.2	Less anxious or fearful	56.7
More articulate/communicative	41.3	Increased motivation	47.2
Improved concentration	40.1	Altered sense of time	37.6
More forgetful	36.5	Enthusiastic	37.3
Difficulty finding words	18.1	Less motivation	23.5
Difficulty concentrating	16.4	Paranoia	14.5
Memory improvement	13.6	Apathetic	8.7
Difficulty making decisions	10.2	Increased anxiety	8.6
Confusion	4.9	Hallucinations	3.8
Long-term memory problems	4.2		
Physiological, %		Movement, %	
Improved sleep	82.1	Desire to clean	40.3
Desire to eat (munchies)	72.7	Desire to stretch/exercise	37.2
Dry mouth	63.0	Desire to be still/couch-lock	31.1
Increased sex drive	48.7	Poor balance/feel unsteady	5.8
Tired/sleepy	45.9	Lack of coordination	5.4
Stimulated/energized	44.4	Artistic/social, %	
Affects dreams	33.9	More creative	72.4
Loss of appetite	9.6	More “inward” focus	50.1
Hurts lungs	7.6	Better social interactions	46.9
Diminished sex drive	6.5	Musical	41.9
Disrupted sleep	5.2	More extraverted, “outward” focus	24.8
Dizziness	5.0	Worse social interactions	12.2
		Less creative	3.4

#### Withdrawal

Participants were asked to use a yes/no scale to report 13 withdrawal symptoms they have experienced with discontinuation of cannabis for ≥72 h. A not applicable (n/a) response option was included for those who had never discontinued cannabis for ≥72 h or who had not experienced any withdrawal symptoms.

#### Addiction

Participants used a yes/no scale to indicate whether they had ever had trouble reducing or stopping their use of cannabis, and they used a yes/no/I don't know scale to indicate whether they believe cannabis is addictive.

### Data analysis

The data were analyzed using IBM SPSS 23. Percentages were computed, and hierarchical logistic regression analyses were used to compare group percentages after statistically controlling for confounding differences in their demographic and cannabis use characteristics. Due to the large number of comparisons and the large sample size, only results with a *p*-value ≤0.001 were considered statistically significant.

## Results

### Characteristics of the cohort

The sample of 2878 cannabis users was classified as “medical” (*n* = 891; 31.0%), “recreational” (*n* = 1110; 38.2%), or “mixed” (selected both medical/recreational; *n* = 877; 30.2%) (Fig. 1). The sample of 2855 individuals who provided their age were further classified by age as young: 18–29 (*n* = 1300; 44.8%); middle: 30–49 (*n* = 1048; 36.1%); or older: 50+ (*n* = 507; 17.5%). Table 1 displays the demographic characteristics and cannabis use patterns of the complete sample. Supplementary Tables S1 and S2 (with consolidated response options) provide this information as a function of user type and age grouping, respectively.

#### Acute effects

Table 2 shows the overall percentage of respondents endorsing each of the acute effects. As shown in the table, the most commonly reported effects (>50% of total) were improved sleep (82.1%), more calm/peaceful (79.7%), desire to eat (72.7%), more creative (72.4%), dry mouth (63%), less anxious/fearful (56.7%), and more “inward” focus (50.1%).

**FIG. 2.**
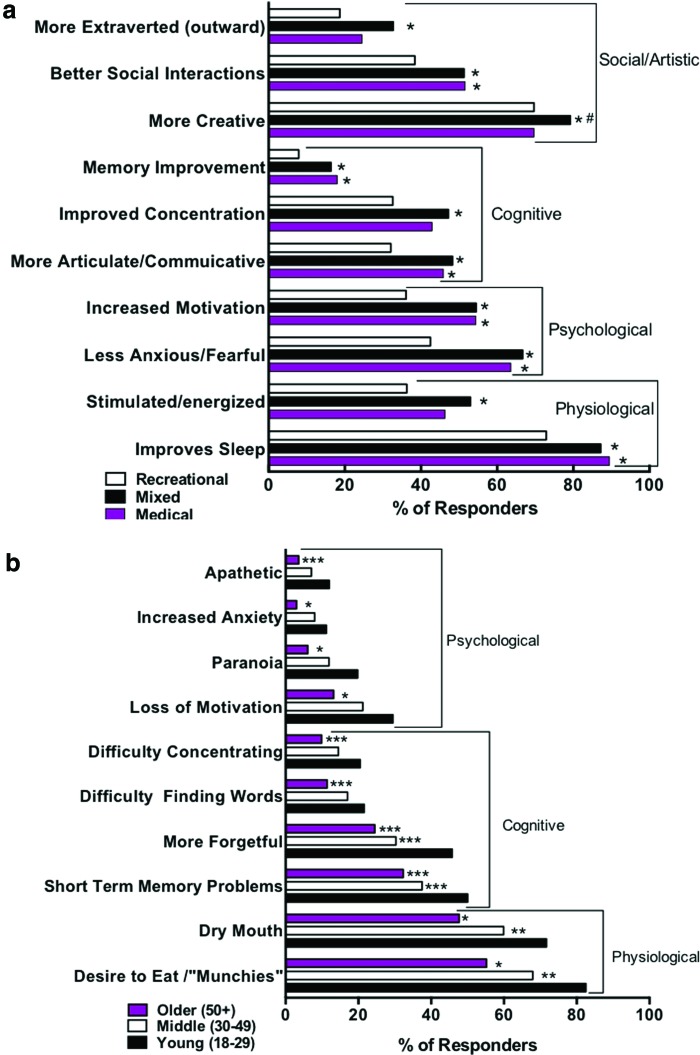
Significant differences in desirable or undesirable acute effects. Bar plots show the raw percentages of each age group selecting acute effects, with significant differences between groups using chi square analyses in reported acute effects. **(a)** Differences in “desirable” acute effects by user type: *p < 0.001 from recreational users; **p < 0.001 from recreational and mixed users; ^#^*p* < 0.001 from medical users. **(b)** Significant differences in “undesirable” acute effects by age grouping. *p < 0.001 from both young and middle age groups; **p < 0.001 from the young and older age groups; ***p < 0.001 from the young age group. Color images are available online.

Supplementary Table S3 shows the percentages of medical, recreational, and “mixed” (medical/recreational) cannabis users who endorsed each of the acute effects. Statistical comparisons of these groups (with the confounding group differences highlighted in Supplementary Table S1 controlled) indicated that medical users were significantly less likely than recreational users to endorse some “undesirable” effects, such as being more forgetful, increased anxiety, and “couch-lock.” Figure 2a shows that medical users were significantly more likely than recreational users to endorse some “desirable” effects: memory improvement, more articulate, less anxious/fearful, increased motivation, improved sleep, and better social interactions.

Further, responders were segregated by age, and it was found that older and middle-age individuals reported fewer undesirable cognitive, psychological, and physiological effects (Supplementary Table S4 and Fig. 2b).

#### Withdrawal symptoms

Overall, 35.2% of responders indicated that withdrawal symptoms were not applicable to them. The most commonly reported withdrawal symptoms were irritability (33.7%), insomnia (30.3%), and anxiety (22.7%). Fewer than 20% of respondents endorsed the remaining withdrawal symptoms (Table 3).

**Table 3.** Percentage of Total Respondents Who Reported Various Withdrawal Symptoms

Withdrawal symptoms, %			
Not applicable	35.2	Tiredness	8.2
Irritability	33.7	Nausea	7.0
Insomnia/interrupted sleep	30.3	Improved productivity	4.8
Anxiety	22.7	Weight loss	3.9
Loss of appetite	18.8	Sweating	3.9
Vivid dreams	17.3	Tremor	1.4
Loss of productivity	12.4	Salivation	0.6
Addictiveness, %			
Is not addictive	68.1	Don't know if addictive	14.8
Is addictive	17.0	Have had trouble stopping	16.7

Survey responders were asked to report yes/no to indicate whether they experience each symptom when removing cannabis for 72 h, or not applicable if they never experience any symptoms or have never removed cannabis for ≥72 h. They could respond yes/no to trouble stopping cannabis and yes/no/don't know to the question about addictiveness.

**FIG. 3.**
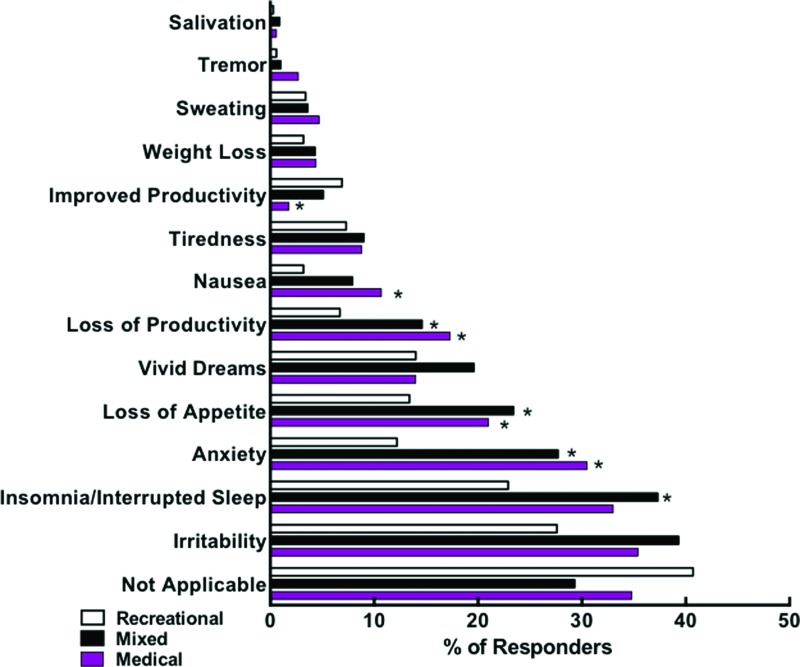
Differential withdrawal effects based on user type: bar graph shows the raw percentages of each user type selecting each effect. Significant differences across groups were determined using *p* ≤ 0.001, after statistically controlling for potentially cofounding group differences: *indicates significant difference from recreational users. Color images are available online.

Comparisons of user types indicated that medical and mixed-type cannabis users reported more undesirable withdrawal symptoms than recreational users. Specifically, as shown in Figure 3, medical and mixed users differed significantly from recreational users with respect to reporting anxiety, loss of productivity, and loss of appetite as symptoms of withdrawal (Supplementary Table S5). Mixed users also reported insomnia more than recreational users. Finally, medical users were significantly more likely to report nausea than recreational users.

Older individuals were significantly less likely than middle-age and younger individuals to endorse several of the withdrawal symptoms, including irritability, insomnia, and loss of appetite (Supplementary Table S6).

#### Addiction

**FIG. 4.**
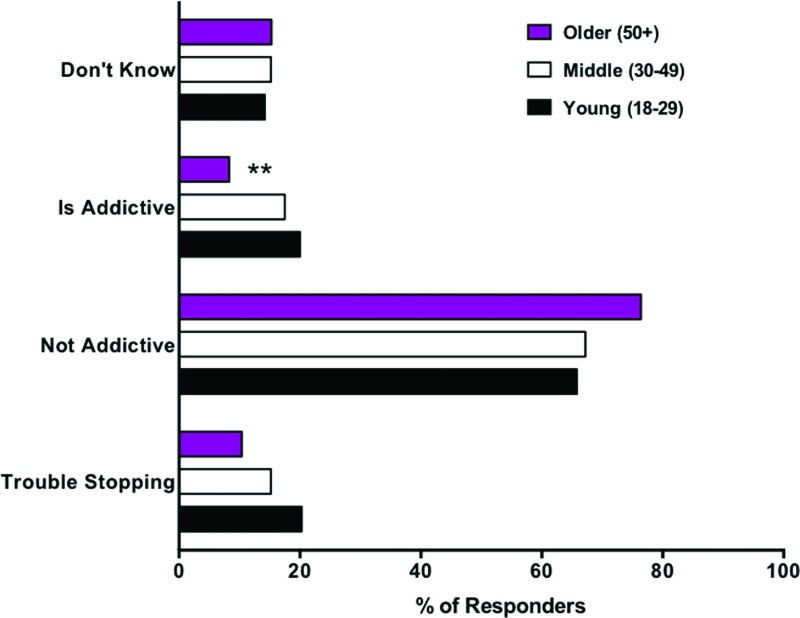
Beliefs about addictiveness among young, middle, and older age groups: bar graph shows the raw percentage of each age group endorsing trouble stopping using cannabis and selecting yes/no/don't know responses to the question “Do you believe that cannabis is addictive?” **Indicates significant difference from both younger and middle-age groups with *p* ≤ 0.001 after statistically controlling for potentially confounding group differences. Color images are available online.

As shown in Table 3, the majority of respondents reported believing that cannabis is not addictive, with only 17% reported believing it is addictive and a similar percentage (16.7%) reporting trouble stopping cannabis. The results of group comparisons revealed that, after controlling for the confounding differences between user types and age groups, a lower percentage of the older cannabis users reported believing that cannabis is addictive than younger and middle-age users (Fig. 4 and Supplementary Tables S5 and S6).

## Discussion

The purpose of this study was to document acute cannabis effects and withdrawal effects as functions of user types and age grouping. Because 91% of this cohort report using inhalation as their primary method of administration, the rapid kinetic and high bioavailability of cannabinoids through this route would predict acute effects to be widely experienced.^48^ In general, the results here mirror acute effects reported in the literature such as desire to eat, dry mouth, and cognitive effects. However, these results expand upon previous research by demonstrating differential effects based on the type of user and age, with medical users and older users reporting fewer undesirable acute effects and recreational users and older users reporting fewer withdrawal symptoms.

Consistent with previous research, the present findings indicate that acute cannabis effects include a degree of cognitive impairment. However, subjective perception of improved cognitive function (including improved concentration [40.1%]; having a sense of clarity/perspective [44.5%]; more articulate/communicative [41.3%]) was high for this entire cohort. Moreover, differential cognitive effects were observed as the older cohort was significantly less likely than the younger individuals to report being forgetful, difficulty concentrating, making decisions, and finding words.

Memory is known to start declining after age 50, and difficulty finding words (a symptom of mild cognitive impairment) is estimated to affect 10%–20% of individuals aging 65+.^49^ Therefore, age differences in reports of undesirable cognitive effects may reflect the fact that older individuals are already experiencing cognitive decline, and thus may be more likely to attribute these symptoms to aging rather than cannabis. Alternatively, changes in the endocannabinoid system across the life span may reflect differential effects of cannabis on the “mature” brain.^50,51^ Specifically, endocannabinoid system activity declines with age.^52^

An alternative interpretation challenges the existing paradigm of negative neurocognitive effects. A chronic low dose of THC showed differential effects in mice, boosting performance in older animals while dropping performance in younger mice.^51^ This low dose regulated CB_1_ gene transcription in a protective and cognitive performance-enhancing manner, and this effect was absent in CB_1_ knockout animals. In addition, THC may have a biphasic effect on cognition, or even procognitive effects associated with acetylcholinesterase inhibition.^53,54^ The older cohort and medical users were significantly more likely to be using CBD, which could indicate that they may have been receiving lower doses of THC compared with other age groups (not a confounder in this analysis).

In addition to reporting fewer undesirable cognitive effects, older individuals were significantly less likely to report increased anxiety, paranoia, apathy, and decreased motivation. Similarly, medical users were more likely than recreational users to endorse feeling less anxious or fearful and increased motivation. The overall high rates of endorsement of “increased motivation” were somewhat surprising. Since a-motivational syndrome was first described in 1972, a fear has been perpetuated that cannabis will sap users of their capacity to function normally in society.^55^ Despite ongoing research on this topic, there is still controversy with some reports finding no differences in global motivation, while other data still support the concept.^56–58^ Nevertheless, the differential effect found between medical/mixed use and recreational use may reflect intention. Medical users are typically seeking improvement in quality of life and increased function, while research suggests that recreational use may be more related to leisure time and relaxation.^17,59^

Overall, the results were equivocal with regard to whether acute cannabis intoxication was associated with feeling tired/sleepy (45.9%) or stimulated/energized (44.4%), with significantly more mixed users than recreational users reporting stimulation and reports of feeling tired/sleepy decreasing as a function of age. While the mechanisms driving these results are currently unclear, they may be attributed to timing of use, type of cannabis used (such as the THC or CBD potency), intent behind use, and/or relief from medical symptoms.^17^ Reports of feeling a lack of coordination or unsteadiness were low, with only 5.8% of responders endorsing this acute effect. Given that poor balance/feeling unsteady can be a symptom of some disease processes or a natural consequence of aging, fall risk is an important consideration. However, note that the older cohort and medical users did not report this symptom with higher frequency.

Collectively, the results reported here indicate that medically compromised and older individuals (who may have more health concerns) may not experience the same neurocognitive consequences of cannabis as recreational users, consistent with another preliminary prospective report.^60^ This may reflect a sense of improved wellness associated with medical cannabis use and/or loss of neurodevelopmental vulnerability associated with some medical conditions and the drugs used to treat them.^61^ The same may hold true for older users who are more likely to have symptoms of aging such as arthralgia, lower back pain, arthritis, dementia, or neurodegeneration. Indeed, an observational retrospective study of cannabis use in patients with Parkinson's disease concluded that overall symptom improvement (82%) was not accompanied by major adverse effects.^62^ Finally, results from this study echo other recent research reporting that medical cannabis use may be associated with improved executive functioning, potentially as a result of reduced symptom burden and improved well-being.^51^ This calls into question whether “impairment” or “intoxication” associated with recreational use is a valid descriptor for medical use. Prospective studies using standardized preparations will need to confirm whether any potential risk of cognitive deficit or other adverse effects outweigh the overall benefits for chronically ill or aging people.

Overall, 35.2% of the total sample reported that withdrawal symptoms were not applicable to them (either because they have never discontinued cannabis use for ≥72 h or because they simply have not experienced any withdrawal effects). For those acknowledging withdrawal effects, older users were less likely to report irritability, insomnia/interrupted sleep, anxiety, and loss of appetite. In contrast, medical/mixed users were significantly more likely to report anxiety, loss of productivity, and loss of appetite than recreational users. The latter symptoms may reflect true withdrawal or could be confounded by a rebound effect (return of medical symptoms treated by cannabis).

The majority of respondents (68.1%) believe that cannabis is not addictive, with older individuals being significantly more likely to report that cannabis is not addictive than younger or middle-age adults. Correspondingly, the percentage of responders reporting trouble stopping cannabis was 16.7%, and did not vary as a function of user type or age. These results are somewhat consistent with previously reported rates of cannabis use disorder (30%) and addiction to cannabis (9%), and suggest that a minority of people do become addicted to cannabis.^63,64^ However, the present finding that 68.1% of respondents indicated believing that cannabis is not addictive is much higher than recent findings that 22.4% of adults in the United States believe that marijuana is not addictive. This discrepancy is likely a function of the different samples surveyed in these two studies (regular cannabis users vs. the general population).^65^

The strengths of this study include the large sample size, which allows for comparison of different types of cannabis users and age cohorts, the assessment of a large number of acute and withdrawal effects, and the anonymous nature of the survey, which reduces socially desirable response. Nevertheless, the study has several limitations due to its observational nature. Since cannabis user type and age were not manipulated, the results only imply associations. The data are self-reported retrospective data, which are subject to recall biases. These data also do not allow for investigation into the effects of different cannabis chemotypes or potencies, and therefore it is unclear whether differences in relative CBD or THC content are driving some of the effects.^66^ They also did not permit for investigation into effects of duration or timing of use. Further, there is likely a self-selection bias: this sample would not reflect effects of individuals who tried cannabis and found the effects intolerable, and thus discontinued use. Similarly, 90% of this cohort were using cannabis on a regular basis (daily or weekly use), and previous research indicates that naïve and regular users may experience different acute effects potentially due to the development of tolerance to some side effects.^12,52–57^ For instance, tolerance to the acute cognitive effects occurs with regular use, and these effects become less prominent when consistent THC dosing is maintained.^67^

## Conclusion

As with most drugs, the acute and withdrawal effects of cannabis are likely time- and dose sensitive, subject to individual variability and environmental effects. As such, it is difficult to draw definitive conclusions about universal effects from these data. Nevertheless, with the rapid expansion of recreational cannabis legalization and an increasing prevalence of use by older individuals, information on the acute and withdrawal effects of cannabis is needed to assist in individual decision making around cannabis use. In addition to providing this information, these data indicate that medical users and older individuals (>50) may experience a more favorable side effect profile than recreational and younger adult users, and that recreational and older users may experience fewer withdrawal symptoms. Future studies need to address potential cognitive benefits of low-dose THC in older adults.

## Supplementary Material

Supplemental data

Supplemental data

Supplemental data

Supplemental data

Supplemental data

Supplemental data
