# Supplementary material for: A Survey of Cannabis Acute Effects and Withdrawal Symptoms: Differential Responses Across User Types and Age
Source: J Altern Complement Med. 2019 Mar 9;25(3):326–35. doi: 10.1089/acm.2018.0319 (PMC6437627; doi:10.1089/acm.2018.0319)
Supplement: Supplemental data [file Supp_Table1.pdf]

Supplementary Data

Supplementary Table S1. Demographic Characteristics and Cannabis Use Patterns Across Cannabis User Types (Medical, Recreational, and Mixed)

	Medical	*Recreational*	*Mixed*	
Age, mean (SEM)	40.88 (0.48)	30.68 (0.35)	34.40 (0.46)	**$F = 150.60$, $p < 0.001$**
Gender (male), %	53.2	59.1	54.8	**$\chi^2 = 30.44$, $p < 0.001$**
Ethnicity (White), %	85.9	85.4	86.4	$\chi^2 = 0.43$, $p = 0.81$
Education, %				
High school/GED or less	28.6	30.2	29.1	$\chi^2 = 0.59$, $p = 0.74$
Technical or associate degree	34.9[a]	18.7[b]	26.9[c]	**$\chi^2 = 66.98$, $p < 0.001$**
Bachelor's degree	22.1[a]	35.3[b]	31.7[b]	**$\chi^2 = 42.06$, $p < 0.001$**
Graduate degree	14.3	15.9	12.3	$\chi^2 = 5.00$, $p = 0.08$
Income, %				
<$20,000	23.4	17.4	20.9	$\chi^2 = 11.03$, $p = 0.004$
$20,000–60,000	39.8	40.1	41.3	$\chi^2 = 0.48$, $p = 0.78$
$60,000–80,000	13.3	10.6	10.7	$\chi^2 = 4.22$, $p = 0.12$
**>**$80,000	23.6[a]	32.0[b]	27.0[a,b]	**$\chi^2 = 17.75$, $p < 0.001$**
Employment, %				
Full-time	44.7[a]	60.1[b]	52.5[c]	**$\chi^2 = 46.44$, $p < 0.001$**
Part-time	16.0[a]	21.0[a,b]	22.8[b]	**$\chi^2 = 13.62$, $p = 0.001$**
Not working	39.2[a]	18.9[b]	24.7[b]	**$\chi^2 = 105.88$, $p < 0.001$**
Relationship status, %				
Married/domestic	56.6[a]	40.1[b]	47.2[b]	**$\chi^2 = 53.84$, $p < 0.001$**
Single	28.4[a]	50.4[b]	40.4[c]	**$\chi^2 = 98.39$, $p < 0.001$**
Other	15.0[a]	9.6[b]	12.4[ab]	**$\chi^2 = 13.72$, $p = 0.001$**
Frequency of use, %				
Daily	74.3[a]	48.1[b]	71.3[a]	**$\chi^2 = 180.43$, $p < 0.001$**
Weekly	20.4[a]	31.4[b]	24.5[a]	**$\chi^2 = 32.44$, $p < 0.001$**
Monthly or less	5.3[a]	20.5[b]	4.2[a]	**$\chi^2 = 113.41$, $p < 0.001$**
Quantity of use, %				
≥1 oz	11.0[a]	2.4[b]	6.1[c]	**$\chi^2 = 61.87$, $p < 0.001$**
1/4 oz	25.1[a]	15.1[b]	23.9[a]	**$\chi^2 = 36.76$, $p < 0.001$**
3–5 g	29.9[a]	26.6[a]	35[b]	**$\chi^2 = 16.35$, $p < 0.001$**
<2 g	34.0[a]	55.9[b]	35.0[a]	**$\chi^2 = 126.42$, $p < 0.001$**
Age of first use, %				
<14	17.0	11.7	15.2	$\chi^2 = 11.81$, $p = 0.003$
14–16	34.8	38.2	37.6	$\chi^2 = 2.65$, $p = 0.26$
17–20	26.0[a]	38.3[b]	34.3[b]	**$\chi^2 = 34.04$, $p < 0.001$**
21+	22.1[a]	11.7[b]	12.9[b]	**$\chi^2 = 46.57$, $p < 0.001$**
Method of use, %				
Inhalation	84.1[a]	96.6[b]	93.8[b]	**$\chi^2 = 110.01$, $p < 0.001$**
Oral	14.3[a]	3.4[b]	5.9[b]	**$\chi^2 = 89.93$, $p < 0.001$**
Method of selection, %				
High THC	40.2[a]	35.2[a]	50.7[b]	**$\chi^2 = 49.49$, $p < 0.001$**
High CBD	48.3[a]	13.6[b]	39.0[c]	**$\chi^2 = 300.27$, $p < 0.001$**
Terpenoids	15.2[a]	3.3[b]	11.2[a]	**$\chi^2 = 85.97$, $p < 0.001$**
Smell	41.0[a]	41.9[a]	49.5[b]	**$\chi2 = 16.05$, $p < 0.001$**

Percentages represent overall raw percentages. Bolded chi-square results indicate an overall significant difference across the three groups. Different superscripts represent specific group differences with $p \leq 0.001$.

CBD, cannabidiol; SEM, standard error of the mean; THC, delta-9 tetrahydrocannabinol.
