# Supplementary material for: A Survey of Cannabis Acute Effects and Withdrawal Symptoms: Differential Responses Across User Types and Age
Source: J Altern Complement Med. 2019 Mar 9;25(3):326–35. doi: 10.1089/acm.2018.0319 (PMC6437627; doi:10.1089/acm.2018.0319)
Supplement: Supplemental data [file Supp_Table2.pdf]

Supplementary Table S2. Demographic Characteristics and Cannabis Use Patterns Across Age Groups

| | *Young, %* | *Middle age, %* | *Older, %* | |
|---|---|---|---|---|
| Gender (male) | 56.7 | 51.3 | 52.3 | $\chi^2 = 7.82$, $p = 0.02$ |
| Ethnicity (White) | 82.2[a] | 88.2[b] | 89.4[b] | **$\chi^2 = 23.64$, $p < 0.001$** |
| Cannabis user type | | | | |
| Medical | 17.8[a] | 37.3[b] | 52.1[c] | **$\chi^2 = 227.85$, $p < 0.001$** |
| Recreational | 49.8[a] | 33.9[b] | 17.8[c] | **$\chi^2 = 170.62$, $p < 0.001$** |
| Mixed | 32.3 | 28.8 | 30.1 | $\chi^2 = 3.48$, $p = 0.17$ |
| Education | | | | |
| High school/GED or less | 37.6[a] | 25.4[b] | 16.5[c] | **$\chi^2 = 90.92$, $p < 0.001$** |
| Technical or associate degree | 22.7[a] | 26.7[a] | 34.7[b] | **$\chi^2 = 27.17$, $p < 0.001$** |
| Bachelor's degree | 33.4[a] | 28.5[a,b] | 25.0[b] | **$\chi^2 = 14.16$, $p = 0.001$** |
| Graduate degree | 6.3[a] | 19.5[b] | 23.8[b] | **$\chi^2 = 128.21$, $p < 0.001$** |
| Income | | | | |
| <$20,000 | 24.5[a] | 15.9[b] | 19.4[a,b] | **$\chi^2 = 26.30$, $p < 0.001$** |
| $20,000–60,000 | 42.4 | 40.0 | 35.9 | $\chi^2 = 6.30$, $p = 0.04$ |
| $60,000–80,000 | 9.0[a] | 13.8[b] | 12.7[a,b] | **$\chi^2 = 13.80$, $p = 0.001$** |
| >$80,000 | 24.0[a] | 30.2[b] | 32.0[b] | **$\chi^2 = 16.39$, $p < 0.001$** |
| Employment | | | | |
| Full-time | 51.7[a] | 64.9[b] | 33.1[c] | **$\chi^2 = 139.40$, $p < 0.001$** |
| Part-time | 27.8[a] | 14.4[b] | 11.3[b] | **$\chi^2 = 92.96$, $p < 0.001$** |
| Not working | 20.4[a] | 20.7[a] | 55.6[b] | **$\chi^2 = 259.05$, $p < 0.001$** |
| Relationship status | | | | |
| Married/domestic | 28.6[a] | 62.8[b] | 65.2[b] | **$\chi^2 = 347.46$, $p < 0.001$** |
| Single | 61.3[a] | 25.9[b] | 16.4[c] | **$\chi^2 = 447.31$, $p < 0.001$** |
| Other | 10.1[a] | 11.3[a] | 18.4[b] | **$\chi^2 = 24.35$, $p < 0.001$** |
| Frequency of use | | | | |
| Daily | 61.5 | 64.1 | 67.5 | $\chi^2 = 5.62$, $p = 0.06$ |
| Weekly | 26.1 | 25.4 | 25.2 | $\chi^2 = 0.22$, $p = 0.89$ |
| Monthly or less | 12.3 | 10.5 | 7.3 | $\chi^2 = 9.45$, $p = 0.009$ |
| Quantity of use, % | | | | |
| ≥1 oz | 4.1[a] | 7.2[b] | 9.9[b] | **$\chi^2 = 23.05$, $p < 0.001$** |
| 1/4 oz | 20.1 | 22.0 | 20.9 | $\chi^2 = 1.25$, $p = 0.53$ |
| 3–5 g | 33.4 | 27.8 | 26.3 | $\chi^2 = 12.50$, $p = 0.002$ |
| <2 g | 42.5 | 43.2 | 42.9 | $\chi^2 = 0.89$, $p = 0.96$ |
| Age of first use | | | | |
| <14 | 12.4 | 17.4 | 13.2 | $\chi^2 = 12.62$, $p = 0.002$ |
| 14–16 | 40.4 | 33.4 | 35.5 | $\chi^2 = 12.71$, $p = 0.002$ |
| 17–20 | 38.0[a] | 28.3[b] | 31.0[a,b] | **$\chi^2 = 26.31$, $p < 0.001$** |
| 21+ | 9.2[a] | 20.9[b] | 20.3[b] | **$\chi^2 = 72.14$, $p < 0.001$** |
| Method of use | | | | |
| Inhalation | 97.0[a] | 90.6[b] | 81.2[c] | **$\chi^2 = 123.35$, $p < 0.001$** |
| Oral | 2.9[a] | 8.5[b] | 17.8[c] | **$\chi^2 = 117.03$, $p < 0.001$** |
| Method of selection | | | | |
| High THC | 44.5[a] | 37.1[b] | 41.4[a,b] | **$\chi^2 = 13.19$, $p = 0.001$** |
| High CBD | 29.5[a] | 31.7[a,b] | 39.4[b] | **$\chi^2 = 16.81$, $p < 0.001$** |
| Terpenoids | 8.6 | 9.7 | 10.1 | $\chi^2 = 1.30$, $p = 0.52$ |
| Smell | 51.9[a] | 40.8[b] | 30.6[c] | **$\chi^2 = 74.44$, $p < 0.001$** |

Percentages represent overall raw percentages. Bolded chi-square results indicate an overall significant difference across the three groups. Different superscripts represent specific group differences with $p \leq 0.001$.

CBD, cannabidiol; THC, delta-9 tetrahydrocannabinol.
