# Supplementary material for: A Survey of Cannabis Acute Effects and Withdrawal Symptoms: Differential Responses Across User Types and Age
Source: J Altern Complement Med. 2019 Mar 9;25(3):326–35. doi: 10.1089/acm.2018.0319 (PMC6437627; doi:10.1089/acm.2018.0319)
Supplement: Supplemental data [file Supp_Table3.pdf]

Supplementary Table S3. Differences in Acute Effects of Cannabis Among Recreational, Medical, and Mixed Users

	Medical (n=*891), %*	*Recreational* (n=*1110), %*	*Mixed* (n=*877), %*	
Cognitive				
More forgetful	26.2[a]	42.0[b]	40.1[b]	**$\chi^2 = 21.92, p < 0.001$**
Short-term memory problems	34.0[a]	45.4[ab]	47.0[b]	**$\chi^2 = 15.74, p < 0.001$**
Long-term memory problems	2.8	5.2	4.2	$\chi^2 = 3.18, p = 0.20$
Memory improvement	18.0[a]	7.9[b]	16.4[a]	**$\chi^2 = 19.91, p < 0.001$**
Difficulty concentrating	11.7	20.1	16.8	$\chi^2 = 3.82, p = 0.15$
Improved concentration	42.9[a,b]	32.6[a]	47.2[b]	**$\chi^2 = 16.75, p < 0.001$**
Difficulty making decisions	13.6	5.8	10.5	$\chi^2 = 9.48, p = 0.009$
Confusion	3.6	5.8	5.0	$\chi^2 = 1.00, p = 0.61$
Sense of clarity/perspective	45.2	40.6	49.0	$\chi^2 = 4.96, p = 0.08$
Difficulty finding words	13.6	21.2	19.0	$\chi^2 = 3.68, p = 0.16$
More articulate/communicative	45.9[a]	32.1[b]	48.3[a]	**$\chi^2 = 28.79, p < 0.001$**
Psychological				
Increased anxiety	4.3[a]	12.8[b]	7.8[a,b]	**$\chi^2 = 14.11, p = 0.001$**
Less anxious or fearful	63.6[a]	43.5[b]	66.7[a]	**$\chi^2 = 73.96, p < 0.001$**
More calm or peaceful	80.5	75.6	84.7	$\chi^2 = 8.77, p = 0.01$
Paranoia	8.3	20.0	13.7	$\chi^2 = 6.06, p = 0.05$
Apathetic	5.8	10.2	9.7	$\chi^2 = 1.69, p = 0.43$
Less motivation	15.7	29.4	24.5	$\chi^2 = 11.59, p = 0.003$
Increased motivation	54.4[a]	36.1[b]	54.4[a]	**$\chi^2 = 23.99, p < 0.001$**
Enthusiastic	34.3[a,b]	34.0[a]	44.8[b]	**$\chi^2 = 13.13, p = 0.001$**
Altered sense of time	29.5[a]	39.5[a]	43.9[b]	**$\chi^2 = 28.84, p < 0.001$**
Hallucinations	3.3	4.8	3.0	$\chi^2 = 1.32, p = 0.52$
Movement				
Poor balance/feel unsteady	5.1	5.9	6.4	$\chi^2 = 3.15, p = 0.16$
Lack of coordination	4.7	5.6	6.2	$\chi^2 = 4.17, p = 0.12$
Desire to be still/couch-lock	24.1[a]	35.0[b]	33.6[b]	$\chi^2 = 8.57, p = 0.01$
Desire to stretch/exercise	37.6	32.7	43.2	$\chi^2 = 7.87, p = 0.02$
Desire to clean	38.3[a,b]	36.3[a]	47.7[b]	**$\chi^2 = 14.12, p = 0.001$**
Physiological				
Tired/sleepy	40.9	46.2	51.0	$\chi^2 = 12.14, p = 0.002$
Disrupts sleep	3.0	6.9	5.1	$\chi^2 = 3.01, p = 0.22$
Improves sleep	89.5[a]	72.9[b]	87.2[a]	**$\chi^2 = 39.98, p < 0.001$**
Affects dreams	25.7	37.3	38.5	$\chi^2 = 11.78, p = 0.003$
Stimulated/energized	46.3[a,b]	36.3[b]	53.0[a]	**$\chi^2 = 18.39, p < 0.001$**
Dry mouth	56.3	65.9	66.2	$\chi^2 = 8.76, p = 0.01$
Dizziness	4.2	5.3	5.4	$\chi^2 = 2.07, p = 0.35$
Diminished sex drive	3.5	8.6	7.1	$\chi^2 = 9.98, p = 0.007$
Increased sex drive	43.7	49.4	53.5	$\chi^2 = 6.17, p = 0.05$
Desire to eat (munchies)	64.2	75.2	77.4	$\chi^2 = 9.82, p = 0.007$
Loss of appetite	8.6	9.5	10.5	$\chi^2 = 0.98, p = 0.61$
Hurts lungs	5.7	10.1	6.4	$\chi^2 = 3.44, p = 0.18$
Artistic and social				
Less creative	2.7	3.2[b]	4.6	$\chi^2 = 3.55, p = 0.17$
More creative	69.7[a]	69.7[a]	79.2[b]	**$\chi^2 = 14.11, p < 0.001$**
Musical	36.0	43.1	46.8	$\chi^2 = 10.24, p = 0.006$
Worse social interactions	7.2	17.7	10.3	$\chi^2 = 10.59, p = 0.005$
Better social interactions	51.6[a]	38.4[b]	53.1[a]	**$\chi^2 = 28.05, p < 0.001$**
More extraverted, ''outward''	24.5[a,b]	18.7[a]	32.7[b]	**$\chi^2 = 28.60, p < 0.001$**
More ''inward'' focus	42.6	54.7	52.3	$\chi^2 = 6.86, p = 0.03$

The results of hierarchical logistic regression analyses used to compare these percentages after controlling for the confounding demographic and cannabis use variables show some significant differences in effects across user types. Percentages represent overall raw percentages (without effects of covariates removed). Bolded chi-square results indicate an overall significant difference ($p < 0.001$) across the three groups with the effects of the covariates (bolded in Supplementary Table S1) statistically removed. Different superscripts represent specific group differences with effects of covariates removed and $p < 0.001$.
