# Supplementary material for: A Survey of Cannabis Acute Effects and Withdrawal Symptoms: Differential Responses Across User Types and Age
Source: J Altern Complement Med. 2019 Mar 9;25(3):326–35. doi: 10.1089/acm.2018.0319 (PMC6437627; doi:10.1089/acm.2018.0319)
Supplement: Supplemental data [file Supp_Table4.pdf]

Supplementary Table S4. Differential Acute Effects of Cannabis Among Young, Middle-Age, and Older Cannabis Users

	Young (n = *1300), %*	*Middle age* (n = *1048), %*	*Older* (n = *507), %*	
Cognitive				
More forgetful	45.8[a]	30.3[b]	24.5[b]	**$\chi^2 = 48.89$, $p < 0.001$**
Short-term memory problems	50.0[a]	37.5[b]	32.3[b]	**$\chi^2 = 32.34$, $p < 0.001$**
Long-term memory problems	5.2	3.7	2.4	$\chi^2 = 2.72$, $p = 0.26$
Memory improvement	13.7	13.5	13.6	$\chi^2 = 0.91$, $p = 0.63$
Difficulty concentrating	20.5[a]	14.5[a,b]	9.9[b]	**$\chi^2 = 13.82$, $p = 0.001$**
Improved concentration	43.7	38.4	36.1	$\chi^2 = 5.83$, $p = 0.05$
Difficulty making decisions	14.2[a]	8.2[a]	4.1[b]	**$\chi^2 = 26.41$, $p < 0.001$**
Confusion	6.2	4.1	3.0	$\chi^2 = 3.14$, $p = 0.21$
Sense of clarity/perspective	45.3	46.0	40.8	$\chi^2 = 4.88$, $p = 0.09$
Difficulty finding words	21.6[a]	17.0[a,b]	11.4[b]	**$\chi^2 = 18.20$, $p < 0.001$**
More articulate/communicative	45.9	32.1	48.3	$\chi^2 = 2.25$, $p = 0.32$
Psychological				
Increased anxiety	11.2[a]	8.0[a]	3.0[b]	**$\chi^2 = 18.43$, $p < 0.001$**
Less anxious or fearful	60.5[a]	55.9[b]	50.1[b]	**$\chi^2 = 27.42$, $p < 0.001$**
More calm or peaceful	82.3	77.9	78.5	$\chi^2 = 2.19$, $p = 0.33$
Paranoia	19.8[a]	11.9[a]	6.1[b]	**$\chi^2 = 32.00$, $p < 0.001$**
Apathetic	12.0[a]	7.1[ab]	3.6[b]	**$\chi^2 = 15.17$, $p = 0.001$**
Less motivation	29.5[a]	21.2[a]	13.2[b]	**$\chi^2 = 21.91$, $p < 0.001$**
Increased motivation	46.0	49.6	46.7	$\chi^2 = 4.09$, $p = 0.13$
Enthusiastic	42.2	34.7	30.8	$\chi^2 = 10.97$, $p = 0.004$
Altered sense of time	44.5[a]	33.4[b]	29.4[b]	**$\chi^2 = 29.04$, $p < 0.001$**
Hallucinations	4.6	3.1	3.2	$\chi^2 = 3.19$, $p = 0.20$
Movement				
Poor balance/feel unsteady	6.9	4.5	5.7	$\chi^2 = 8.85$, $p = 0.01$
Lack of coordination	6.5	5.0	3.6	$\chi^2 = 10.36$, $p = 0.006$
Desire to be still/couch-lock	39.7[a]	26.5[b]	18.7[c]	**$\chi^2 = 36.42$, $p < 0.001$**
Desire to stretch/exercise	40.5[a]	37.6[a]	29.0[b]	**$\chi^2 = 17.98$, $p < 0.001$**
Desire to clean	44.5[a]	41.6[a]	27.6[b]	**$\chi^2 = 25.05$, $p < 0.001$**
Physiological				
Tired/sleepy	55.2[a]	40.9[b]	32.9[c]	**$\chi^2 = 61.30$, $p < 0.001$**
Disrupts sleep	5.8	5.6	2.8	$\chi^2 = 6.97$, $p = 0.03$
Improves sleep	81.6	81.3	86.0	$\chi^2 = 1.47$, $p = 0.48$
Affects dreams	43.0[a]	30.0[b]	19.5[c]	**$\chi^2 = 49.09$, $p < 0.001$**
Stimulated/energized	43.8	45.1	44.6	$\chi^2 = 0.23$, $p = 0.89$
Dry mouth	71.7[a]	59.9[b]	47.7[c]	**$\chi^2 = 38.91$, $p < 0.001$**
Dizziness	5.6	3.8	5.3	$\chi^2 = 2.38$, $p = 0.30$
Diminished sex drive	8.9[a]	5.0[b]	3.6[b]	**$\chi^2 = 18.14$, $p < 0.001$**
Increased sex drive	56.3[a]	46.8[b]	33.9[c]	**$\chi^2 = 45.53$, $p < 0.001$**
Desire to eat (munchies)	82.5[a]	67.9[b]	55.2[c]	**$\chi^2 = 73.72$, $p < 0.001$**
Loss of appetite	10.5	9.4	7.7	$\chi^2 = 1.06$, $p = 0.59$
Hurts lungs	8.4	7.4	5.7	$\chi^2 = 3.01$, $p = 0.22$
Artistic and social				
Less creative	3.8	3.2	2.6	$\chi^2 = 1.56$, $p = 0.46$
More creative	75.4	71.4	68.2	$\chi^2 = 0.77$, $p = 0.68$
Musical	48.5	38.6	32.9	$\chi^2 = 5.76$, $p = 0.06$
Worse social interactions	15.9	10.2	6.5	$\chi^2 = 10.31$, $p = 0.006$
Better social interactions	50.2[a]	46.6[a]	39.8[b]	**$\chi^2 = 30.39$, $p < 0.001$**
More extraverted, "outward"	26.5	24.0	22.9	$\chi^2 = 4.07$, $p = 0.13$
More "inward" focus	56.1	47.4	40.8	$\chi^2 = 11.30$, $p = 0.004$

The results of hierarchical logistic regression analyses used to compare these percentages after controlling for the confounding demographic and cannabis use variables show some significant differences in effects across age groups. Percentages represent overall raw percentages (without effects of covariates removed). Bolded chi-square results indicate an overall significant difference ($p \leq 0.001$) across the three groups with the effects of the covariates (bolded in Supplementary Table S2) statistically removed. Different superscripts represent specific group differences with effects of covariates removed and $p \leq 0.001$.
