# Supplementary material for: A Survey of Cannabis Acute Effects and Withdrawal Symptoms: Differential Responses Across User Types and Age
Source: J Altern Complement Med. 2019 Mar 9;25(3):326–35. doi: 10.1089/acm.2018.0319 (PMC6437627; doi:10.1089/acm.2018.0319)
Supplement: Supplemental data [file Supp_Table5.pdf]

Supplementary Table S5. Differences in Withdrawal Symptoms and Beliefs About Addictiveness of Cannabis Among Recreational, Medical, and Mixed Cannabis Users

	Medical (n = *891), %*	*Recreational* (n = *1110), %*	*Mixed* (n = *877), %*	
Withdrawal symptoms				
Not applicable	34.8	40.7	29.3	$\chi^2 = 6.25$, $p = 0.04$
Anxiety	30.5[a]	12.2[b]	27.7[a]	**$\chi^2 = 67.88$, $p < 0.001$**
Irritability	35.4	27.6	39.3	$\chi^2 = 9.70$, $p = 0.008$
Insomnia/interrupted sleep	33.0[a,b]	22.9[b]	37.3[a]	**$\chi^2 = 18.88$, $p < 0.001$**
Tiredness	8.8	7.3	9.0	$\chi^2 = 6.57$, $p = 0.04$
Vivid dreams	14.0	14.0	19.6	$\chi^2 = 2.35$, $p = 0.31$
Loss of productivity	17.3[a]	6.7[b]	14.6[a]	**$\chi^2 = 32.77$, $p < 0.001$**
Improved productivity	1.8[a]	6.9[b]	5.1[a,b]	**$\chi^2 = 15.85$, $p < 0.001$**
Nausea	10.7[a]	3.2[b]	7.9[a,b]	**$\chi^2 = 20.98$, $p < 0.001$**
Loss of appetite	21.0[a]	13.4[b]	23.4[a]	**$\chi^2 = 24.77$, $p < 0.001$**
Weight loss	4.4	3.2	4.3	$\chi^2 = 5.60$, $p = 0.06$
Tremor	2.7	0.6	1.0	$\chi^2 = 12.40$, $p = 0.002$
Sweating	4.7	3.4	3.6	$\chi^2 = 5.04$, $p = 0.08$
Salivation	0.6	0.3	0.9	$\chi^2 = 4.41$, $p = 0.11$
Addictiveness				
Trouble stopping	13.8	18.8	16.6	$\chi^2 = 4.14$, $p = 0.13$
Cannabis is addictive	12.7	21.9	15.2	$\chi^2 = 6.71$, $p = 0.03$
Cannabis not addictive	71.7	64.2	69.8	$\chi^2 = 4.98$, $p = 0.08$
Don't know if addictive	15.6	13.9	15.0	$\chi^2 = 0.52$, $p = 0.77$

Percentages represent overall raw percentages (without effects of covariates removed). Bolded chi-square results indicate an overall significant difference ($p \leq 0.001$) across the three groups with the effects of the covariates (bolded in Supplementary Table S1) statistically removed. Different superscripts represent specific group differences with effects of covariates removed and $p \leq 0.001$.
