# Supplementary material for: A Survey of Cannabis Acute Effects and Withdrawal Symptoms: Differential Responses Across User Types and Age
Source: J Altern Complement Med. 2019 Mar 9;25(3):326–35. doi: 10.1089/acm.2018.0319 (PMC6437627; doi:10.1089/acm.2018.0319)
Supplement: Supplemental data [file Supp_Table6.pdf]

Supplementary Table S6. Differences in Withdrawal Symptoms and Beliefs About Addictiveness of Cannabis Among Young, Middle-Age, and Older Cannabis Users

	Young (n = *1300*), %	*Middle age* (n = *1048*), %	*Older* (n = *507*) %	
Withdrawal symptoms				
Not applicable	30.2[a]	36.3[a]	47.1[b]	**$\chi^2 = 28.38$, $p < 0.001$**
Irritability	35.2[a]	37.5[a]	22.5[b]	**$\chi^2 = 30.02$, $p < 0.001$**
Insomnia/interrupted sleep	33.5[a]	30.4[a]	22.7[b]	**$\chi^2 = 16.43$, $p < 0.001$**
Anxiety	22.8[ab]	25.8[a]	16.4[b]	**$\chi^2 = 19.92$, $p < 0.001$**
Loss of appetite	26.3[a]	15.8[b]	5.9[c]	**$\chi^2 = 96.72$, $p < 0.001$**
Vivid dreams	21.9	15.6	9.7	$\chi^2 = 12.09$, $p = 0.002$
Loss of productivity	12.8	13.2	9.5	$\chi^2 = 7.31$, $p = 0.03$
Tiredness	10.1	7.3	5.5	$\chi^2 = 3.66$, $p = 0.16$
Nausea	8.0[a]	7.0[a,b]	4.7[b]	**$\chi^2 = 13.57$, $p = 0.001$**
Improved productivity	7.2	3.1	2.0	$\chi^2 = 8.17$, $p = 0.02$
Weight loss	5.7[a]	2.8[a,b]	1.0[b]	**$\chi^2 = 23.04$, $p < 0.001$**
Sweating	5.1	3.4	1.8	$\chi^2 = 10.68$, $p = 0.005$
Tremor	1.5	1.1	1.6	$\chi^2 = 1.58$, $p = 0.45$
Salivation	0.9	0.3	0.2	$\chi^2 = 4.88$, $p = 0.09$
Addictiveness				
Trouble stopping	20.3	15.2	10.4	$\chi^2 = 9.52$, $p = 0.009$
Cannabis is addictive	20.0[a]	17.5[a]	8.3[b]	**$\chi^2 = 25.62$, $p < 0.001$**
Cannabis is not addictive	65.8	67.2	76.4	$\chi^2 = 11.87$, $p = 0.003$
Don't know if it's addictive	14.2	15.2	15.3	$\chi^2 = 0.75$, $p = 0.69$

Percentages represent overall raw percentages (without effects of covariates removed). Bolded chi-square results indicate an overall significant difference ($p \leq 0.001$) across the three groups with the effects of the covariates (bolded in Supplementary Table S2) statistically removed. Different superscripts represent specific group differences with effects of covariates removed and $p \leq 0.001$.
